# Combined transcortical endoscopic and open approach for resection of pediatric craniopharyngioma

**DOI:** 10.3171/2026.7.FOCVID26120

**Published:** 2026-10-01

**Authors:** Casey A. Jarvis, Lissa C. Baird

**Affiliations:** 1 Department of Neurosurgery, Brigham and Women’s Hospital, Boston; and; 2 Department of Neurosurgery, Boston Children’s Hospital, Boston, Massachusetts

**Keywords:** pediatric, craniopharyngioma, endoscopic, orbitozygomatic

## Abstract

This combined transcortical endoscopic and open approach for resection of pediatric craniopharyngioma minimizes parenchymal traction during open resection. A flexible endoscope is passed through Kocher’s point into the ipsilateral lateral ventricle. The Fogarty balloon and irrigation are used to dissect the capsule away from the hypothalamus and other surrounding anatomical structures. Once the cyst is free and decompressed, resection is completed using a subfrontal approach through the opticocarotid triangle via a modified orbitozygomatic craniotomy.

The video can be found here: https://stream.cadmore.media/r10.3171/2026.7.FOCVID26120

## Transcript

Combined transcortical endoscopic and open approach for resection of pediatric craniopharyngioma. Craniopharyngiomas are rare suprasellar tumors comprising about 6%–9% of all CNS tumors in the pediatric population.^1^ Although they are benign pathologies, due to their location of origin and tendency to adhere to surrounding structures, they are associated with significant treatment-related endocrine and hypothalamic morbidity.^2,3^ Many grading systems have thus been developed to characterize the location of the tumor or its relationship to the optic chiasm, pituitary stalk, or hypothalamus, in order to guide management.^4–8^

### 0:58 Treatment Options.

The mainstay of treatment for craniopharyngioma is surgical resection, whether that be an open or endoscopic endonasal approach. Recent studies have demonstrated that EEA may offer improved visual outcomes and rates of gross-total resection, although they do carry a higher risk of CSF leak and are limited for tumors with significant lateral extension.^9,10^

Radiation can be employed either alone or as an adjuvant treatment for residual postoperatively. New medical therapies have also emerged, taking advantage of tumor biology, with inhibitors targeting the BRAF V600E mutation in papillary craniopharyngioma and WNT inhibitors targeting the CTNNB1 mutation in adamantinomatous craniopharyngioma, although the latter remain largely in the preclinical phase.^10^

### 1:44 Treatment Options: Novel Strategy.

To address these issues, we propose a novel surgical strategy: the combined transcortical endoscopic and open approach for resection of pediatric craniopharyngiomas—a two-staged approach involving transcortical ventricular endoscopy to dissect the tumor capsule free from the hypothalamus, followed by a cranio-orbitozygomatic or endoscopic endonasal approach for definitive resection, depending on the anatomy of the tumor.

### 2:05 Key Equipment.

The key equipment required for this approach include a flexible and rigid endoscope, a Fogarty catheter and balloon, and the NICO Myriad system, which is a microcutting and aspiration device.

### 2:17 Explanation of the Approach.

For the endoscopic portion of this approach, Kocher’s point is chosen for burr hole entry and EVD placement. Once CSF return is obtained, the EVD is removed and the flexible scope is passed through the tract. The Fogarty balloon and irrigation are used in combination with the flexible endoscope to gently dissect the tumor capsule from the surrounding structures. This can then be exchanged for a rigid scope for tumor debulking.

Once the cyst capsule is freed and decompressed, we move on to the open segment of the approach. A frontotemporal incision is made and a one-piece modified cranio-orbitozygomatic craniotomy is completed with a matchstick drill and osteotomes. A subfrontal transsylvian approach provides access to the tumor, which is debulked using the NICO Myriad system.

### 2:59 Case 1: Presentation.

A 5-year-old previously healthy female presented to an outside hospital ED with 2 weeks of vomiting and strabismus. She was found to have a large, complex cystic and solid suprasellar mass with calcifications that extended into the third ventricle, prepontine space, and cerebellopontine angles bilaterally, concerning for a craniopharyngioma. Due the extension of the mass in the intra- and extraventricular space, it was characterized as Yaşargil type D. Vision was intact, although papilledema was present, and no endocrine function deficits were noted.

### 3:34 Case 1: Presentation Continued.

The patient was treated at the outside hospital with cyst drainage and left frontal Ommaya placement with good resolution of her presenting symptoms. A postprocedural MRI demonstrates significant decrease in the size of the cyst and improvement in hydrocephalus. The patient was treated with serial taps for a period of 6 months until it was noted that the third ventricular portion of the cyst seemed to be increasing in size, indicating it was no longer in communication with the prepontine space where the Ommaya catheter terminated. The patient then presented to Boston Children’s Hospital for definitive surgical management. Due to the size of the tumor and significant involvement with the surrounding structures, the combined transcortical endoscopic and open approach was chosen in order to free the cyst from above first and prevent undue traction on the fornices and hypothalamus during the removal of the tumor from below.

### 4:25 Case 1: Positioning.

A left-sided approach was planned. A frontotemporal incision was mapped extending past midline to allow for removal of the patient’s existing Ommaya reservoir, outlined here, and access to Kocher’s point for the initial endoscopic segment of the approach. The patient was positioned supine with the head turned to the right. Intraoperative navigation and MRI were used.

### 4:45 Case 1: Endoscopic Portion.

This video begins shortly after entry into the left lateral ventricle with the flexible endoscope. Here, we can observe the left foramen of Monro framing our cyst capsule beneath. We can appreciate the significant adherence of the tumor to the fornix as well as the wall of the hypothalamus. The stiff Bugbee electrode is used without cautery to create a plane between the tumor capsule and the fornix. We can also appreciate the ostomy left in the floor of the third ventricle by the Ommaya catheter. Next, the Fogarty balloon is used to gently dissect along the capsule of the cyst in order to free the cyst from the hypothalamic wall and ependyma. The expansion of the balloon provides gentle traction and dissection along natural planes. The cyst capsule is then punctured and the contents are aspirated. The cyst cavity is explored. The decompressed cyst is now freed from the hypothalamic walls, and the flexible endoscope is exchanged for a rigid scope for tumor debulking. Endoscopic microforceps are used to grasp the tumor capsule; a gentle twisting motion frees small fragments of the tumor. This technique is repeated until the tumor is debulked, as we can see here at the conclusion of the endoscopic segment of the procedure.

### 6:38 Case 1: Open Portion.

The dura is opened in a C-shaped fashion. The sylvian fissure is split with both sharp and blunt dissection. The tumor capsule is revealed within the fissure. The capsule is punctured, and the contents are aspirated. The membrane is resected, revealing more solid components of the tumor. Next, the tumor is dissected carefully off of the left optic nerve. More loculated components of the cyst are opened and aspirated. Tumor is dissected carefully off of the internal carotid artery to open the opticocarotid triangle. Tumor resection proceeds by a combination of electrocautery and suction, blunt dissection, and sharp dissection. Here, we can see the optic chiasm coming into view. The NICO is also employed for further debulking of solid tumor. The last bits of tumor are carefully dissected from the optic chiasm and perforators. Finally, a small angled mirror is employed to check the underside of the chiasm and any other blind spots for residual.

### 8:01 Case 1: Postoperative Course.

Postoperative imaging demonstrated excellent resection with some expected residual enhancement along the anterior hypothalamic wall due to invasion in this location. The patient woke up at her neurological baseline, with intact vision and cognition, but with DI and panhypopituitarism treated with hormone replacement therapy. At 4–6 months’ follow-up, she began to have a mild increase in BMI treated with metformin and nutritional modifications.

### 8:27 Case 2: Presentation.

An 11-year-old male with no significant past medical history presented with 2 months of progressive intermittent headaches. His symptoms acutely worsened over 48 hours with associated nausea and vomiting and subjective blurry vision, prompting the family to seek medical care urgently. MRI was significant for a large heterogeneously enhancing, cystic, calcified suprasellar mass, concerning for craniopharyngioma, with significant ventriculomegaly. Ophthalmology evaluation at the time demonstrated subtle optic disc edema and full visual fields. Similarly to our first case, this tumor has both intraventricular and extraventricular extension, classifying as a Yaşargil type D. He was noted to have low IGF-1 for his age, with otherwise normal endocrine function. While the anatomy of this case is also favorable for an endoscopic endonasal approach, a transcortical endoscopic approach was chosen due to concern for significant hypothalamic adherence, with a combined open approach per surgeon preference.

### 9:26 Case 2: Endoscopic Portion.

Here, we can see our classic anatomical landmarks seen from the endoscopic approach including the choroid plexus, septal vein, and thalamostriate vein framing the foramen of Monro with our cyst capsule beneath. We gently dissect along the capsule of the cyst using the flexible endoscope with the Fogarty balloon and irrigation, in order to free the cyst from the overlying fornices, hypothalamic wall, and ependyma. The cyst is then punctured and the contents are aspirated, and we see our decompressed cyst here at the end.

### 9:57 Case 2: Open Portion.

Here, we can see the left optic nerve and internal carotid artery forming the opticocarotid triangle. The tumor is gently resected through this triangle with a combination of electrocautery, suction, and sharp and blunt dissection.

### 10:12 Case 2: Postoperative Course.

Postoperative imaging demonstrated excellent resection with no observed residual or surgical complications. The patient was neurologically intact postoperatively with intact vision, but did have DI and panhypopituitarism. He has not had any issues with weight gain or hypothalamic obesity and has had no evidence of tumor recurrence in 18 months of follow-up.

## Disclosures

The authors report no conflict of interest concerning the materials or methods used in this study or the findings specified in this publication.

## Author Contributions

Primary surgeon: Baird. Assistant surgeon: Jarvis. Editing and drafting the video and abstract: both authors. Critically revising the work: both authors. Reviewed submitted version of the work: both authors. Approved the final version of the work on behalf of both authors: Baird. Supervision: Baird.

## Supplemental Information

### Previous Presentations

Portions of this work were presented at the North American Skull Base Society Annual Meeting, New Orleans, LA, February 13–16, 2025.

### Patient Informed Consent

The necessary patient informed consent was obtained in this study.
